# Advancing decision-making in shock wave lithotripsy for upper ureteral stones: the role of radiological stone impaction markers

**DOI:** 10.1007/s00240-025-01797-y

**Published:** 2025-07-17

**Authors:** Marcin Popiolek, Johan Jendeberg, Max Olin, Magnus Wagenius, Pernilla Sundqvist, Mats Lidén

**Affiliations:** 1 Department of Urology, Faculty of Medicine and Health, Örebro University, Örebro, Sweden; 2 Department of Radiology, Faculty of Medicine and Health, Örebro University, Örebro, Sweden; 3 Department of Surgery, Blekinge Hospital, Karlskrona, Sweden; 4 Department of Urology, Helsingborg Hospital, Helsingborg, Sweden; 5 Department of Urology, Örebro University Hospital, Örebro, 701 85 Sweden

**Keywords:** Shock wave lithotripsy, Ureteral stone, Inflammation, Impaction Markes

## Abstract

This work aims to evaluate whether radiological signs of stone impaction (RSSI) measured on non-contrast computed tomography (CT) can predict shock wave lithotripsy (SWL) outcomes for upper ureteral stones and to assess whether integrating these markers into an existing prediction model (the Niwa nomogram) improves predictive performance. We retrospectively analysed 256 patients treated with SWL for upper ureteral stones between 2012 and 2019. Standard stone parameters and RSSI, including ureteral wall thickness (UWT), ureteral diameters and CT attenuations above and below the stone, were assessed. Multivariable logistic regression, receiver operating characteristic (ROC) analysis, net reclassification improvement (NRI) and decision curve analysis (DCA) were used to evaluate predictive performance. The Niwa nomogram was enhanced by incorporating significant RSSI parameters and was internally validated using *k*-fold cross-validation. Maximum ureteral attenuation below the stone (UABSmax), ureter diameter above the stone (UDAS) and renal pelvis diameter (RPD) were found to be associated with SWL outcome. UABSmax had the highest individual predictive value (area under the curve (AUC) 0.66), while UWT showed no significant association or predictive value. Incorporating UABSmax and RPD into the Niwa nomogram (Niwa+) marginally increased AUC (0.72 vs. 0.71) but did not lead to significant improvements in NRI or DCA. In conclusion, certain RSSI– particularly UABSmax and RPD– were associated with SWL outcome but provided limited value when added to an already validated nomogram.

## Introduction

Urolithiasis places a significant burden on both affected individuals and healthcare systems. While small ureteral stones often pass spontaneously and can be managed conservatively [1], there is no clear consensus on optimal treatment strategies, particularly regarding intervention timing and choice of treatment modality. The European Association of Urology (EAU) Guidelines recommend early intervention for ureteral stones unlikely to pass spontaneously [2]. Notably, upper ureteral stones are recognised as having a lower probability of spontaneous passage (22–48%) and often necessitate intervention [1, 3]. Managing these stones can be complex, requiring sometimes repeated procedures, which may lead to complications and compromise patients’ quality of life.

The EAU guidelines endorse either shock wave lithotripsy (SWL) or ureteroscopy (URS) as first-line treatment options for ureteral stones smaller than 10 mm due to their comparable outcomes [2]. SWL, being less invasive and usually performed without general anaesthesia, remains an attractive option. However, its success is influenced by more than just stone size, including anatomical location, density, internal composition, and skin-to-stone distance (SSD) [4–7]. Therefore, appropriate patient selection is required to optimize SWL outcomes.

To support clinical decision-making, various scoring systems and nomograms have been developed and validated, including the Niwa nomogram, which has performed promisingly in predicting SWL success for upper ureteral stones, as confirmed by a recent validation study [8, 9]. However, emerging evidence suggests that factors indicating inflammation and impaction around the stone, such as ureteral wall thickness (UWT) or grade of dilatation of the ureter above the stone may also influence SWL outcomes [10–14]. Given the limited evidence, it remains unclear whether radiological signs of stone impaction measured on non-contrast computed tomography (NCCT) provide additional predictive value beyond that of the well-established predictors.

To address this gap, this study aims to assess whether radiological signs of stone impaction (RSSI) such as UWT, ureter diameters and attenuations below and above the stone, and renal pelvis diameter (RPD) serve as independent predictors of single-session SWL outcome in patients with proximal ureteral stones. Furthermore, the study explores whether incorporating these parameters into the Niwa nomogram may improve its predictive performance. By refining SWL selection criteria, our findings may contribute to more personalised treatment strategies and improved patient outcomes.

## Materials and methods

### Study population

Ethical approval was obtained from the Swedish Ethical Review Authority (Ref. No. 2019–04689). The study used a database described in a recently published validation study [9], which included detailed patient-related, stone-related and SWL outcome data but did not include RSSI. Our study also complies with the transparent reporting of multivariate predictive models on individual prognosis and diagnosis (TRIPOD) statement [15].

**Fig. 1:**
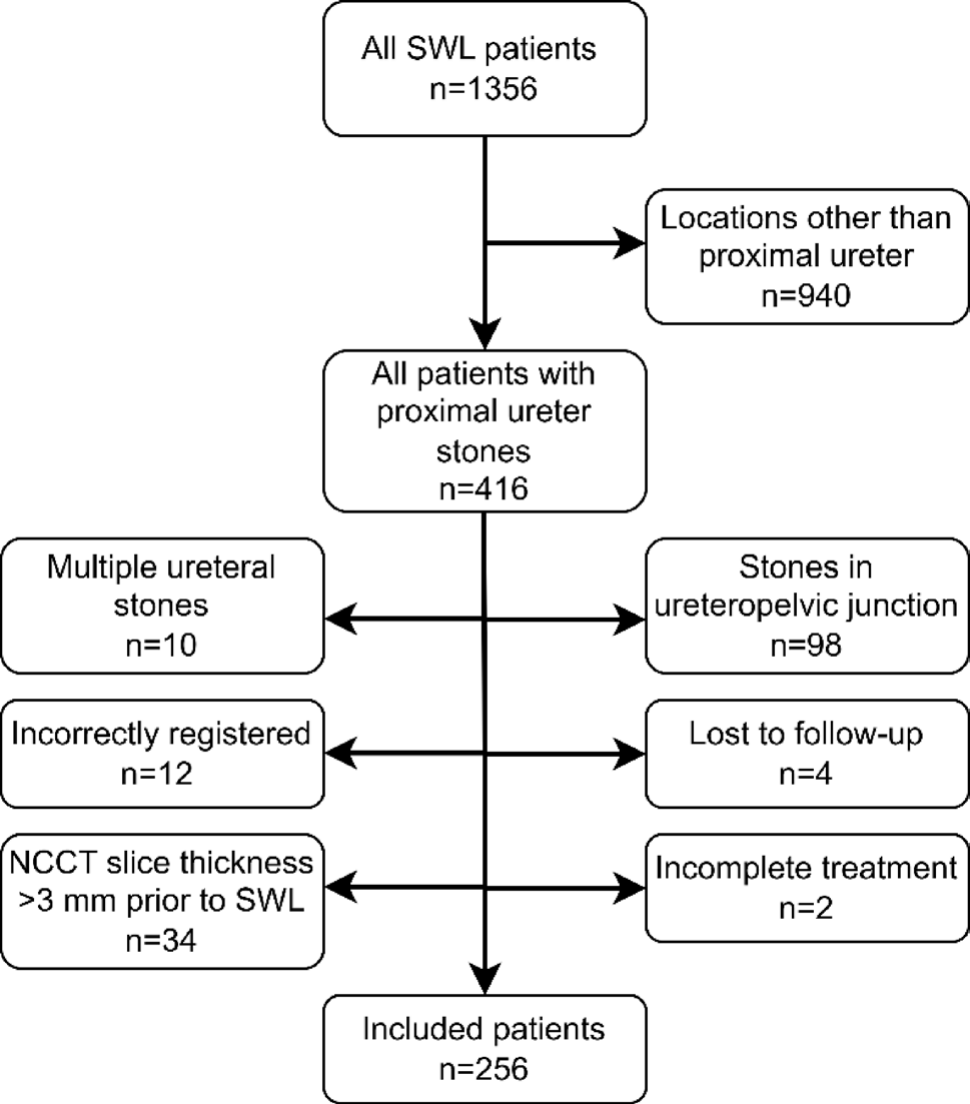
Flowchart showing exclusion criteria with numbers

A total of 1353 patients treated with SWL at the Örebro University Hospital between January 2012 and December 2019 were retrospectively reviewed. After applying exclusion criteria, 256 patients were included in the final cohort. Details on exclusion criteria and the number of excluded patients are outlined in Fig. 1.

## Sample size considerations

The sample size was designed to balance statistical rigor with feasibility, given the retrospective nature of the study. Data from an internal audit indicated a success rate of approximately 60–70% for single-session SWL in proximal stones. This rate was considered sufficient to ensure an adequate number of events (i.e. successful outcomes) and non-events (i.e. unsuccessful outcomes), facilitating robust predictive modelling and internal validation.

## CT scans and image review

All included patients underwent computed tomography (CT) prior to SWL. Only patients with NCCT scans with an axial plane slice thickness ≤ 3 mm were included in this analysis. The CT examinations typically followed a low-dose protocol using 120 kV and a CT dose index (CTDI) between 2 and 5 mGy, in accordance with the institutional imaging standards.

Stone-related measurements were conducted by a single experienced urologist (MP), according to the methodology described in detail in a previous study [9]. Stone dimensions were measured in axial, coronal, and sagittal planes using a soft-tissue window (L50/W400), with the maximum diameter across all planes recorded as the stone length. Stone attenuation was measured according to the level of the stone’s largest axial diameter by placing a circular region of interest (ROI) manually within 2/3 of the stone’s surface (on average) and outside it (maximum). SSD was measured from the stone centre to the skin surface in both a perpendicular (90°) and oblique (45°) orientation on axial slices. RPD was measured on axial images as the maximum distance between the anterior and posterior walls of the renal pelvis. Hydronephrosis was graded on a four-point scale from 0 to 3, where 0 indicated no dilatation, 1 mild, 2 moderate, and 3 severe hydronephrosis.

## Radiological signs of stone impaction

**Fig. 2:**
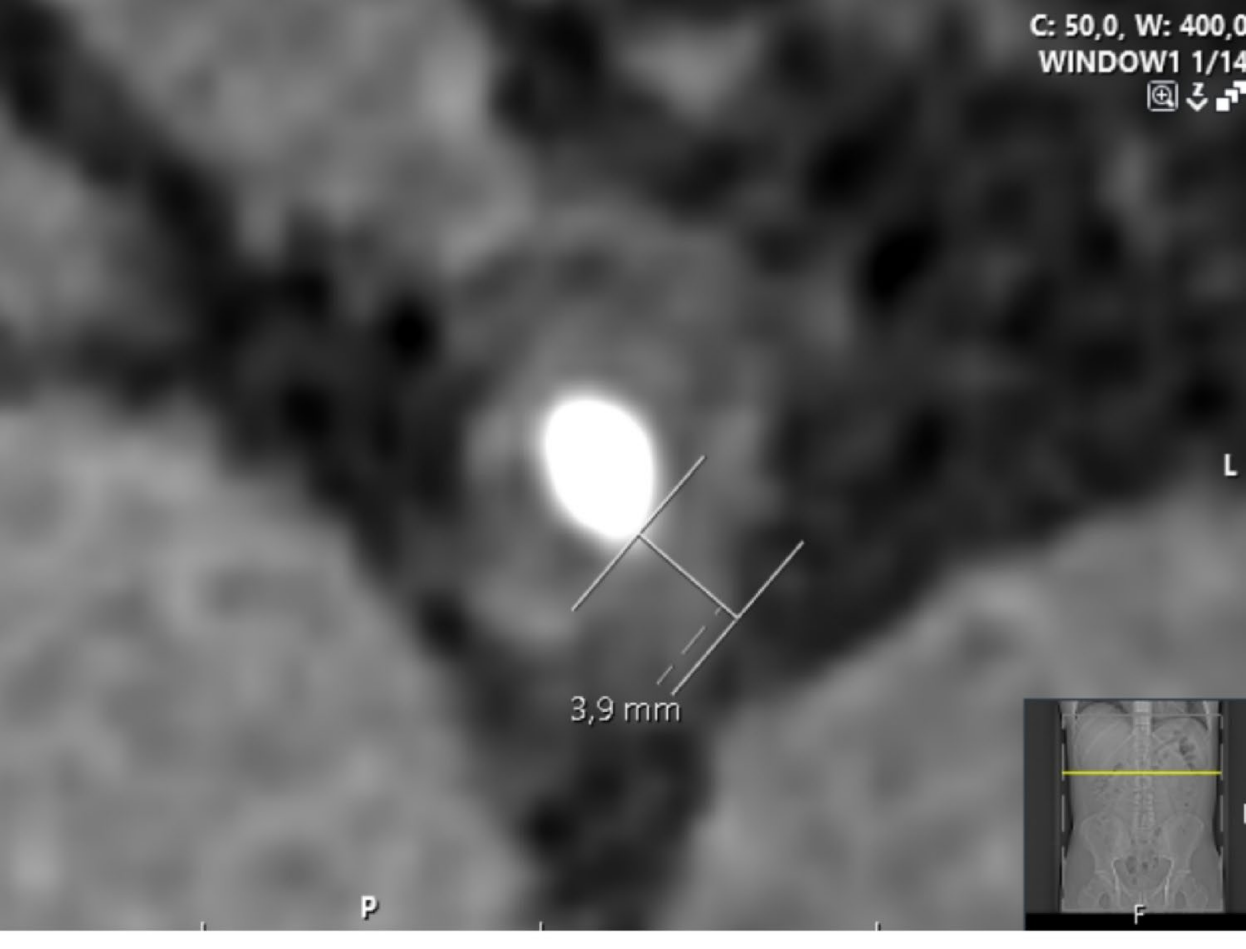
Ureteral wall thickness measured on a 3-mm axial slice

**Fig. 3:**
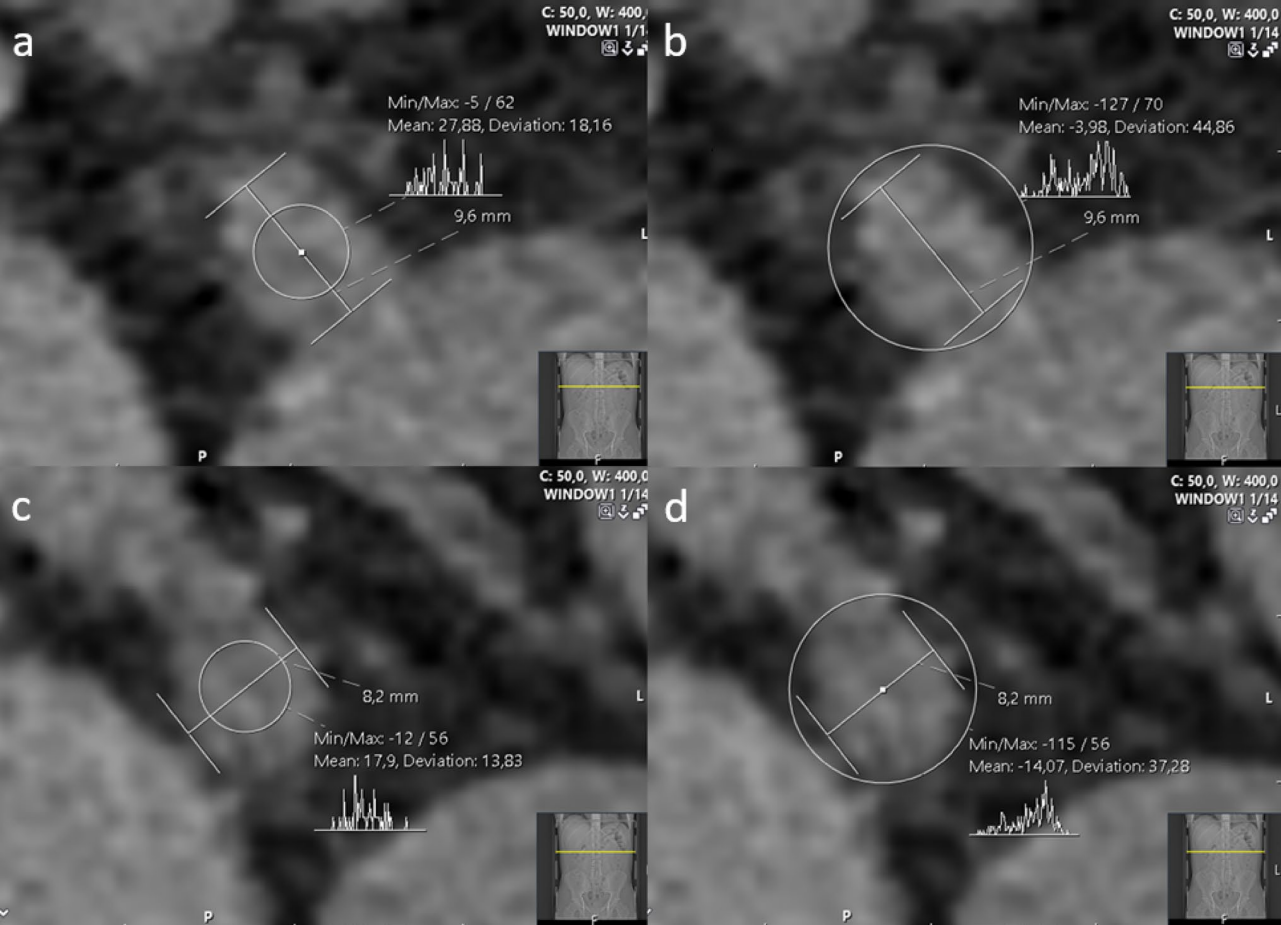
Ureter diameters and attenuations above (**a**,** b**) and below (**c**,** d**) the stone. In this example, the UAASmean, UAASmax and UDAS were 28 HU, 70 HU and 9.6 mm, respectively, and the UABSmean, UABSmax and UDBS were 18 HU, 56 HU and 8.2 mm, respectively

RSSI were assessed by three independent readers (MP, JJ, MO), who were blinded to the SWL outcomes. These measurements were consistent with the protocol described by Popiolek et al. [16], with the exception that all measurements were performed exclusively on axial reformats in a standardised soft-tissue window (L50/W400), as shown in Figs. 2 and 3. The median value from all readers was used for further analysis.

The following parameters were recorded:

- *UWT* measured at the point of maximal soft tissue thickening at the level corresponding to the stone’s largest axial diameter (Fig. 2);
- *Ureteral diameters above and below the stone* (UDAS and UDBS), measured at the widest place above and below the stone (Fig. 3);
- *Average and maximum attenuation values above and below the stone* (UAASmean UAASmax, UABSmean, UABSmax), measured at the same place as the ureter diameters by placing a circular ROI within 2/3 of the ureter surface (average) and widening it outside the ureter (maximum) (Fig. 3).

The proximal ureter was defined as the portion extending from the ureteropelvic junction to the level where the ureter overlaps with the superior margin of the sacroiliac joint. Stones in the ureteropelvic junction protruding in the renal pelvis were excluded.

## SWL protocol

As previously described [9], SWL procedures were carried out using the Siemens Lithostar Modularis (Siemens AG, Erlangen, Germany) under fluoroscopic control, with patients positioned supine. During the procedure, intermittent small doses of intravenous alfentanil and propofol were administered to provide analgesia and sedation. Adhering to the local protocol, stones located at the level of kidney parenchyma were treated with a maximum energy of 4 kV and up to 4000 shockwaves. Stones located below the parenchyma were treated with a maximum energy of 6 kV and up to 2500 shockwaves. All treatments were delivered at a frequency of 60 impulses per minute (1 Hz).

The outcome measure for SWL performance in the present study– that is, stone-free status– was defined as a complete absence of ureteral calculi at follow-up examination after a single SWL session. Follow-up imaging to evaluate treatment outcomes was conducted 2–6 weeks after the initial treatment, depending on the presumed results. The imaging modalities included plain abdominal X-ray of the kidneys, ureters and bladder (KUB) (*n* = 13); antegrade pyelogram (*n* = 4); conventional excretory urography (*n* = 37); and CT (*n* = 202).

### The Niwa nomogram

As reported in a recent validation study, the Niwa nomogram demonstrated acceptable discrimination, with an area under the curve (AUC) of 0.71 compared with 0.76 in the original study [9]. It outperformed other models in terms of calibration accuracy and provided the highest net benefit. The nomogram’s design incorporates stone length (mm), maximum stone CT attenuation (Hounsfield units) and SSD measured at 90° (cm).

## Statistics

Differences between groups (successful vs. failed SWL) were assessed using the Mann–Whitney U test for non-normally distributed continuous variables and Student’s *t*-test for normally distributed continuous variables. The Chi-square test or Fisher’s exact test was applied for categorical variables, as appropriate. Collinearity between variables was assessed using correlation matrices and the variance inflation factor (VIF), with a VIF threshold of < 5 being considered acceptable to exclude multicollinearity. We applied multivariable logistic regression models to explore independent associations with treatment outcome. Odds ratios (ORs) with 95% confidence intervals (CIs) were reported.

A receiver operating characteristic (ROC) curve analysis was performed to assess the discriminatory ability of the predictive variables, with the AUC used as a measure of model performance. Probabilities for the Niwa nomogram were calculated using the beta coefficients published in the original study. Additional RSSI variables were subsequently incorporated into the Niwa nomogram using logistic regression. To assess the impact of adding RSSI to the original model, internal validation was performed via *k*-fold cross-validation (*k* = 5). Model performance was further compared using NRI and DCA to determine clinical utility. All statistical analyses were conducted with Stata MP 18.0 (StataCorp, Texas, USA), with a significance threshold set at *P* < 0.05.

## Results

Baseline clinical data are summarised in Table 1. A total of 256 patients were included, of whom 166 (65%) had successful SWL. Patients in the failed SWL group were older (64.0 vs. 54.7 years, *P* = 0.002), predominantly male (88% vs. 71%, *P* = 0.002) and had higher creatinine levels (91.0 vs. 79.0 mmol/L, *P* = 0.001).

**Table 1:** Baseline demographic and clinical characteristics of the study population, stratified by SWL outcome

			All patients (*n* = 256)	Successful SWL (*n* = 166)	Failed SWL (*n* = 90)	*P* value
Age (yrs): median (IQR)			58.0 (45.0–67.0)	54.7 (44.0–64.0)	64.0 (51.0–68.8)	0.002
Sex: n (%)						
		Male	197 (77%)	118 (71%)	79 (88%)	0.002
		Female	59 (23%)	48 (29%)	11 (12%)	
Side: n (%)						
		Right	116 (45%)	85 (51%)	31 (34%)	0.01
		Left	140 (55%)	81 (49%)	59 (66%)	
BMI: mean ± SD			28.1 ± 4.5	27.9 ± 4.6	28.4 ± 4.4	0.4
Creatinine (mmol/L): median (IQR)			83.0 (71.0–100.0)	79.0 (68.0–93.0)	91.0 (78.0–111.0)	0.001
Hydronephrosis (%)						0.001
	None		38 (15%)	29 (17%)	9 (10%)	
	Grade 1		156 (61%)	109 (66%)	47 (52%)	
	Grade 2		51 (20%)	25 (15%)	26 (29%)	
	Grade 3		11 (4%)	3 (2%)	8 (89%)	
Stone-free, n (%)			166 (65%)			

*IQR* Interquartile range, *BMI* body mass index, *SD* Standard deviation

In the univariate analysis, stone-related factors significantly associated with SWL failure included larger stone size (8.9 vs. 7.5 mm, *P* < 0.001), higher mean (1053 vs. 909 HU, < 0.001) and maximal stone CT attenuation (1312 vs. 1181 HU, *P* < 0.001). Anatomical differences were also observed, with greater SSD at 90° (13.5 vs. 13.0 cm, *P* = 0.049) and 45° (14.4 vs. 13.9 cm, *P* = 0.044) in the failed group (Table 2). Additionally, ureteral and renal pelvis parameters, such as larger ureter diameter above the stone (UDAS: 10.1 vs. 9.1 mm, *P* = 0.003) and higher ureteral attenuation below the stone (UABSmax: 102 vs. 85 HU, *P* < 0.001), were significantly associated with SWL failure (Table 2).

**Table 2:** Comparison of clinical and radiological parameters between patients with successful and failed SWL outcomes

		All patients (*n* = 256)	Successful SWL (*n* = 166)	Failed SWL (*n* = 90)	*P* value
Stone length (mm): median (IQR)		7.8 (6.4–9.6)	7.5 (6.2–8.6)	8.9 (7.4–10.6)	< 0.001
Mean CT stone attenuation (HU): mean ± SD		960 ± 255	909 ± 244	1053 ± 250	< 0.001
Maximal CT stone attenuation (HU): mean ± SD		1227 ± 284	1181 ± 276	1312 ± 280	< 0.001
SSD (cm): mean ± SD					
	At 90°	13.2 ± 1.9	13.0 ± 1.9	13.5 ± 2.0	0.049
	At 45°	14.1 ± 2.0	13.9 ± 2.0	14.4 ± 2.1	0.044
UWT (mm): mean ± SD		2.4 ± 1.1	2.4 ± 1.0	2.4 ± 1.3	0.9
UDAS (mm): median (IQR)		9.4 (8.0–11.0)	9.1 (7.8–10.3)	10.1 (8.4–11.9)	0.003
UAASmean (HU): median (IQR)		16.6 (9.9–24.1)	18.1 (9.9–25.1)	15.6 (9.8–22.2)	0.2
UAASmax (HU): median (IQR)		98.5 (77.5–122.0)	95.5 (77.0–111.0)	104.0(84.0–132.0)	0.004
UDBS (mm): mean ± SD		6.9 ± 1.9	6.8 ± 1.9	6.9 ± 1.8	0.7
UABSmean (HU): median (IQR)		23.1 (15.8–31.9)	22.6 (15.2–31.2)	25.3 (16.8–33.5)	0.3
UABSmax (HU): median (IQR)		90.0 (73.5–109.0)	85.0 (68.0–103.0)	102.0(84.0–126.0)	< 0.001
RPD (mm): median (IQR)		17.1 (12.8–22.1)	16.4 (12.0–21.0)	19.0 (14.0–24.0)	0.003

*IQR* interquartile range, *HU* Hounsfield units, *SD* standard deviation, *CT* computed tomography, *UWT* ureter wall thickness, *UDAS* ureter diameter above the stone, *UAASmean* ureter mean CT attenuation above the stone, *UAASmax* ureter maximum CT attenuation above the stone, *UDBS* ureter diameter below the stone, *UABSmean* ureter mean CT attenuation below the stone, *UABSmax* ureter maximum CT attenuation below the stone, *RPD* renal pelvis diameter

### Multivariable logistic regression

Multivariable logistic regression revealed that a greater stone length (*P* = 0.04) and larger RPD (*P* = 0.04) were associated with treatment failure. Among RSSI, a smaller ureter diameter below the stone (*P* = 0.01) and a higher maximum ureteral attenuation below the stone (*P* < 0.001) were significantly associated with treatment failure. Other variables, including sex, age, stone side, creatinine levels and other RSSI, did not show significant associations (Table 3).

**Table 3:** Multivariable logistic regression analysis for predictors of outcome. The table presents β coefficients, ors, 95% cis, and *P* values for various clinical and imaging parameters

	β coefficient	OR	95% CI		*P*
Sex (m ref)	0.72	2.10	0.88	4.84	0.1
Age	−0.02	0.98	0.96	1.00	0.09
Side (left ref)	−0.66	0.52	0.27	0.99	0.05
Creatinine	−0.004	0.99	0.98	1.01	0.5
Stone length	−2.0	0.81	0.67	0.99	0.04
Mean CT stone attenuation	−0.001	0.99	0.99	1.00	0.06
SSD at 90°	−0.007	0.99	0.97	1.01	0.42
RPD	−0.05	0.95	0.91	0.99	0.04
UWT	0.21	1.24	0.88	1.75	0.22
UDAS	−0.17	0.84	0.69	1.03	0.08
UAASmax	0.003	1.00	0.99	1.02	0.65
UDBS	0.31	1.36	1.08	1.71	0.01
UABSmax	−0.02	0.98	0.97	0.99	< 0.001

*OR* odds ratio, *CI* confidence interval

### ROC analysis of RSSI

**Fig. 4:**
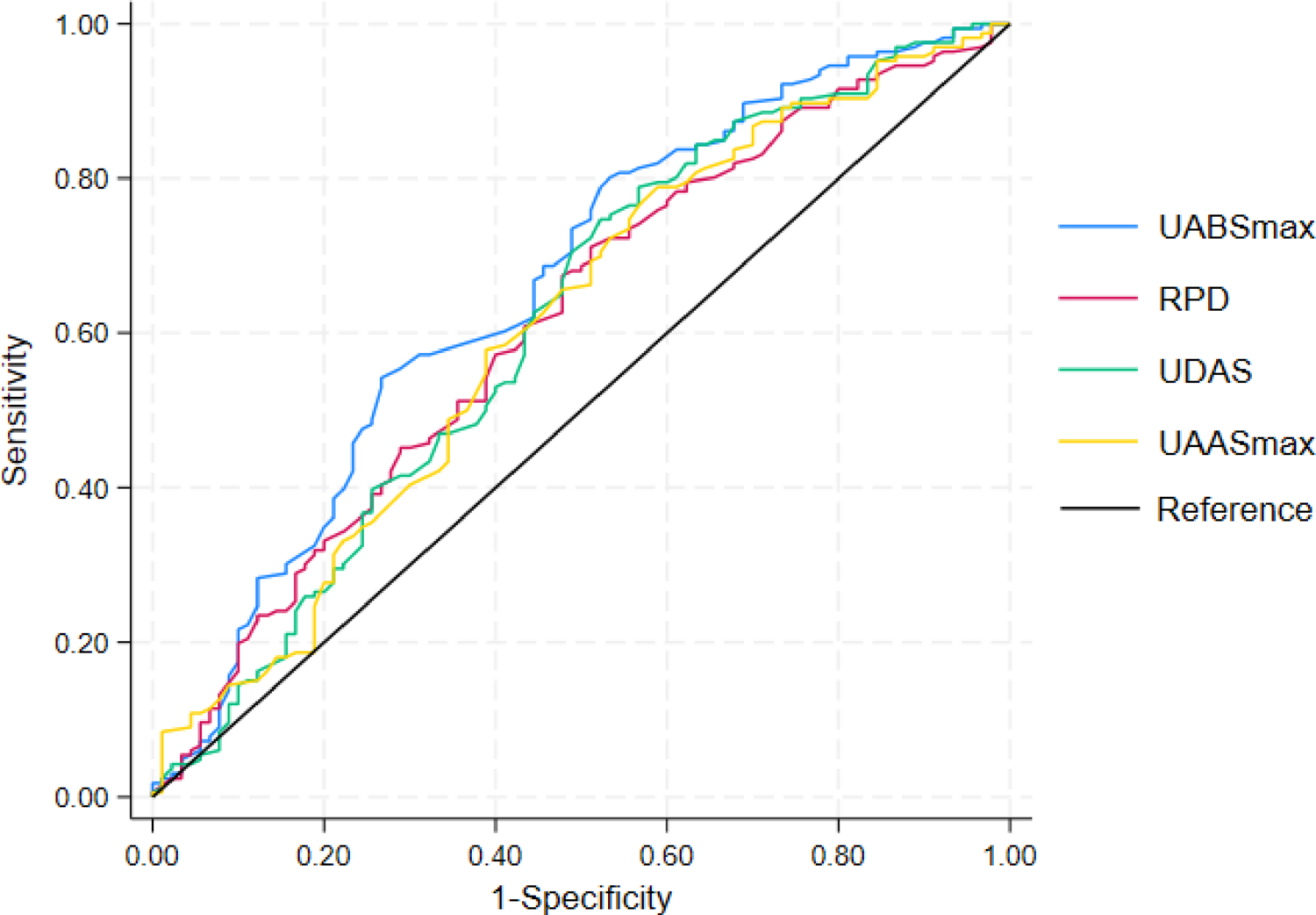
ROC curves for SWL outcome. The explanatory variables are UABSmax, RPD, UDAS and UAASmax; the *y*-axis represents sensitivity, and the *x*-axis shows 1-specificity

The ROC analysis (Fig. 4; Table 4) showed that UABSmax had the highest predictive value for SWL success (AUC 0.66). RPD and UDAS also demonstrated moderate discrimination (AUC 0.61), while UWT and UDBS had the lowest predictive ability (AUC 0.52), indicating limited value in outcome prediction.

**Table 4:** Predictive performance of individual RSSI parameters for SWL outcome

	AUC	95% CI	
UABSmax	0.66	0.59	0.73
RPD	0.61	0.54	0.69
UDAS	0.61	0.54	0.69
UAASmax	0.61	0.53	0.68
UAASmean	0.55	0.48	0.62
UABSmean	0.54	0.46	0.62
UWT	0.52	0.44	0.59
UDBS	0.52	0.44	0.59

### Nomogram enhancements and internal validation

**Fig. 5:**
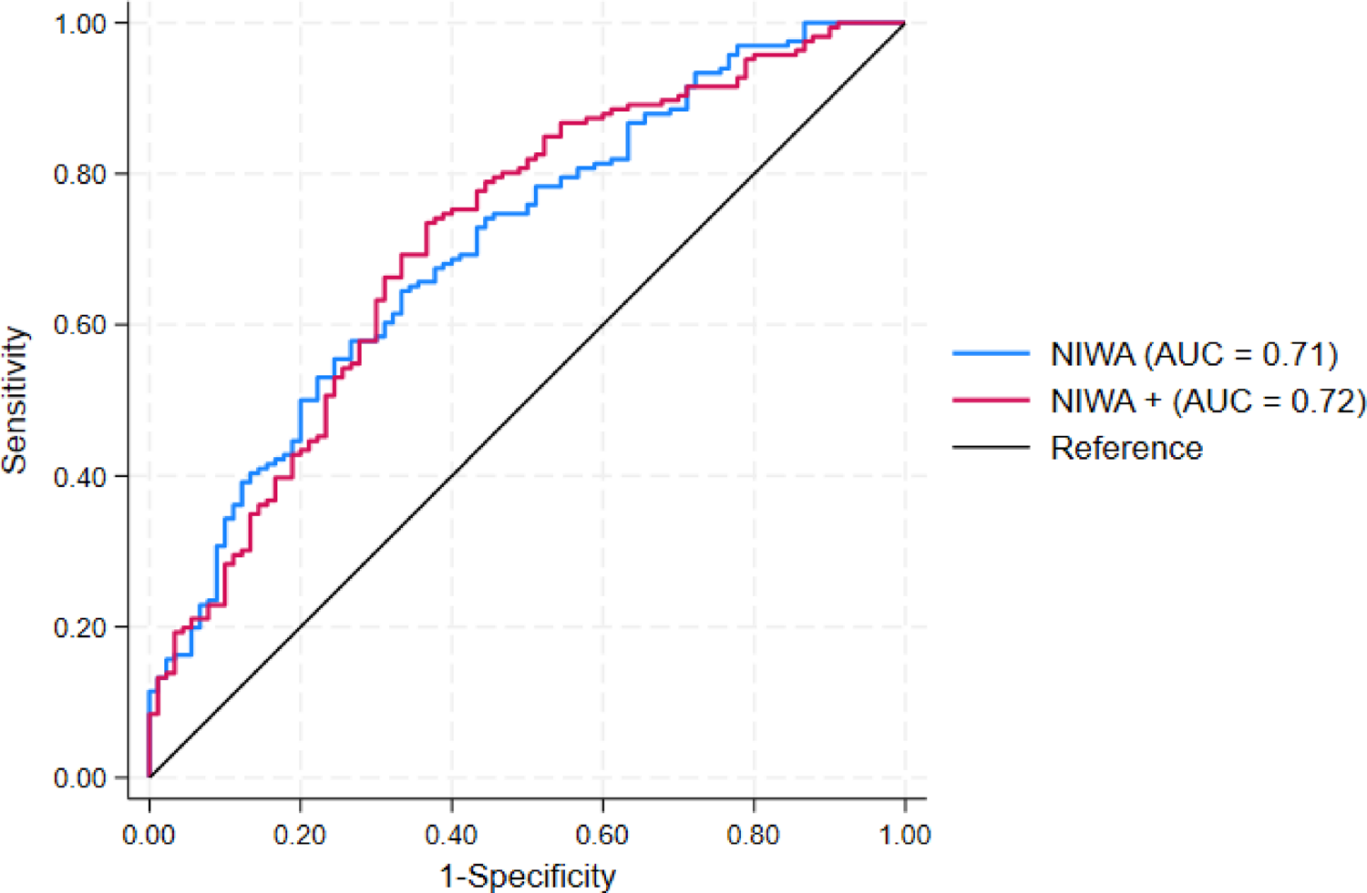
ROC curves for the Niwa nomogram and its enhanced version, the Niwa + nomogram, with RPD and UABSmax

As UABSmax and RPD were identified as significant predictors in the multivariable analysis, they were incorporated into the Niwa nomogram. When assessed individually, they also demonstrated moderate discriminatory capacity (AUCs of 0.66 and 0.61, respectively). The improved model (Niwa+) was internally validated using *k*-fold cross-validation, leading to an averaged AUC of 0.72 (95% bias-corrected CI: 0.65–0.79). However, based on the cross-validated fitted probabilities, the Niwa + nomogram did not show a statistically significant improvement in predictive ability compared with the original model (AUC of 0.71 vs. 0.72, *P* = 0.7) (Fig. 5).

### Net reclassification improvement

The NRI was calculated to evaluate whether the updated model (Niwa+) correctly reclassifies individuals into more appropriate risk categories, using predefined risk thresholds: <0.2 (low, SWL not recommended), 0.2–0.8 (medium, SWL can be considered) and > 0.8 (high, SWL recommended). The overall NRI was 0.08 (95% bias-corrected CI: −0.06–0.22, *P* = 0.2), indicating a small (8%) and non-significant improvement in patient reclassification.

### Decision curve analysis

**Fig. 6:**
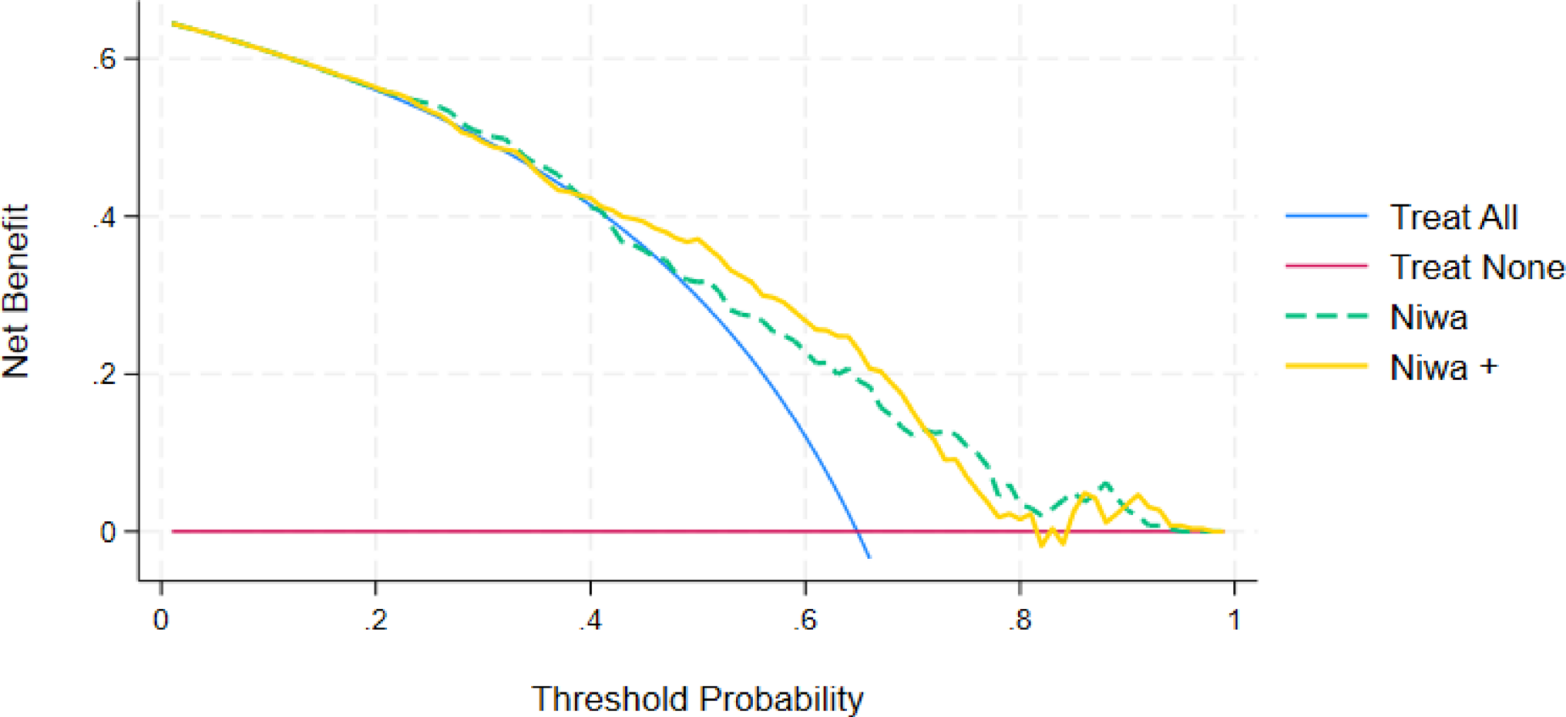
The decision curve analysis (DCA): net benefit

A DCA was performed to assess the clinical utility of the improved Niwa + nomogram, but it revealed only marginal improvement over the original Niwa model. While Niwa + showed a slightly higher net benefit in the 0.2–0.7 threshold probability range, its performance declined for thresholds above 0.7, where it underperformed compared with the original model (Fig. 6).

## Discussion

In this retrospective study, we evaluated whether RSSI measured on NCCT can predict the outcomes of single-session SWL for upper ureteral stones. In summary, the ureteral attenuation below the stone and renal pelvis diameter were statistically significantly associated with SWL outcome, but they only marginally increased the discriminatory effect of the Niwa nomogram (AUC of 0.72 vs. 0.71). Although not independently predictive in our cohort, age was significantly higher in the failed SWL group. Previous studies reported reduced SWL success in older patients, possibly due to age-related tissue changes and delayed fragment clearance [5, 17–19].

Multiple studies have shown that increased UWT is a strong predictor of SWL failure. Reported cut-off values vary, ranging from > 1.82 mm to > 5.25 mm, but all support the association between higher UWT and reduced success [10, 11]. Sarica et al. and Guler et al. respectively highlighted UWT as an independent risk factor for treatment failure in proximal and impacted ureteral stones [13, 14]. Interestingly, Yamashita et al. found that ureteral wall volume was an even stronger predictor than UWT, suggesting that volumetric assessment may more accurately reflect stone impaction grade [20]. A recently published systematic review and metanalysis by Dean et al. focused on evaluating UWT in the context of SWL [12]. Their pooled analysis demonstrated that a thinner UWT was significantly associated with successful SWL. However, they also emphasised the lack of standardised measurement protocols and noted considerable heterogeneity across studies. In our previous study, which remains the only study to date that specifically evaluates the interobserver variability of UWT, to our knowledge, we found that UWT measurements on NCCT showed only moderate inter-reader reliability (ICC = 0.63) and high variability, with limits of agreement of ± 2.0 mm between observers [16]. Although earlier publications have highlighted increased UWT as a reliable predictor of unsuccessful SWL, we were unable to confirm these findings in our cohort. In contrast, we observed no significant difference in UWT between success and failure groups, and the predictive performance of UWT was poor (AUC = 0.52), indicating no discriminatory capability in our dataset.

Several factors may explain this discrepancy. First, methodological inconsistencies– such as differences in CT slice thickness, imaging planes, definition of outcome or the absence of standardised protocols across the studies– could have influenced the measurement outcomes, potentially affecting the predictive accuracy. Second, the timing of imaging relative to symptom onset may affect UWT values, as inflammation may subside over time. Finally, it is possible that UWT is not a reliable predictor in all populations, and previous findings may not be generalisable across settings. These considerations highlight the need for standardised assessment methods and further external validation.

We also evaluated additional CT-based markers potentially associated with stone impaction, including renal pelvis diameter, as well as ureteral diameters and attenuations both above and below the stone. The univariate analysis showed that high UABSmax, RPD and UDAS were significantly associated with SWL failure. Whereas UABSmax measured the attenuation of soft tissues below the stone, with high values possibly indicating a collapsed ureter with secondary inflammatory changes, UDAS captured a dilated ureter, likely reflecting a distended ureter lumen together with an inflamed wall due to obstruction caused by an impacted stone. Of these three markers, UABSmax demonstrated the highest individual predictive value (AUC = 0.66), suggesting that inflammatory changes distal to the stone may play a role in treatment outcome. In contrast, UDBS showed no discriminatory ability (AUC = 0.52), indicating limited value in our cohort. To our knowledge, this is the first study to report the prognostic value of ureteral diameter and attenuation below the stone in the context of SWL, although prior studies by Özbir et al. and Deguchi et al. independently showed that attenuation values proximal and distal to the stone may reflect the degree of impaction [21, 22].

Given these findings, we explored whether incorporating UABSmax and RPD into the existing Niwa nomogram could improve its predictive performance. Although both variables were statistically significant in the multivariable analysis and showed moderate discriminatory capacity individually, the enhanced model (Niwa+) demonstrated only a marginal increase in AUC (from 0.71 to 0.72) following internal cross-validation. Moreover, the NRI and DCA showed no significant clinical advantage of the updated model over the original. These results suggest that, while novel RSSI such as UABSmax and RPD may capture additional aspects of stone impaction, their overall contribution to clinical decision-making remains limited when added to an already relatively well-performing model.

This study has several limitations. First, the retrospective design may have introduced selection bias and unmeasured confounding. However, we minimised this risk through strict inclusion criteria. Second, while interobserver variability in RSSI measurements was not formally assessed, all measurements were performed by experienced, blinded readers using a predefined protocol, which likely reduced subjectivity. Third, the study was conducted at a single tertiary centre, and findings may not be generalisable to other institutions with different imaging protocols, SWL techniques or patient populations. Conversely, this factor also ensured consistency in imaging techniques, SWL equipment and treatment protocols, strengthening internal validity. Finally, the relatively limited sample size– especially within the failure group– may have reduced the power to detect smaller effects or confirm less prominent predictors. However, the sample was sufficiently powered for the primary analyses and allowed for internal validation.

In conclusion, certain radiological signs of stone impaction, particularly maximum ureter CT attenuation below the stone and renal pelvis diameter, were found to be significantly associated with SWL outcomes. However, their integration into an already validated nomogram resulted in no clinically significant improvement in predictive performance compared with the model based on stone size, attenuation and skin-to-stone distance alone.

## Data Availability

The datasets generated and/or analysed during the current study are not publicly available due to current data protection legislation, but are available from the corresponding author on reasonable request, if appropriate permits are obtained from adequate authorities.

## Abbreviations

SWL: Shock wave lithotripsy
RSSI: Radiological signs of stone impaction
UWT: Ureteral wall thickness
UDAS: Ureter diameter above the stone
UDBS: Ureter diameter below the stone
UAAS: Ureter attenuation above the stone
UABS: Ureter attenuation below the stone
HU: Hounsfield units
NCCT: Non-contrast enhanced computed tomography
